# Host-induced gene silencing targeting the calcineurin of *Fusarium fujikuroi* to enhance resistance against rice bakanae disease

**DOI:** 10.3389/fpls.2025.1366158

**Published:** 2025-04-14

**Authors:** Yi-Hsuan Hou, Ting-Xiang Zhang, Ying-Lien Chen

**Affiliations:** ^1^ Department of Plant Pathology and Microbiology, National Taiwan University, Taipei, Taiwan; ^2^ Institute of Biotechnology, National Taiwan University, Taipei, Taiwan; ^3^ Agricultural Biotechnology Research Center, Academia Sinica, Taipei, Taiwan; ^4^ Master Program for Plant Medicine, National Taiwan University, Taipei, Taiwan

**Keywords:** *Fusarium fujikuroi*, calcineurin, *Oryza sativa*, host-induced gene silencing, bakanae disease

## Abstract

Bakanae, or foolish seedling disease of rice, is caused by the ascomycetous fungus *Fusarium fujikuroi*, which is prevalent in many rice-growing countries. Current protection strategies depend on fungicides, but this results in chemical-resistant *F. fujikuroi* and detrimental environmental effects. It is known that calcineurin controls Ca^2+^ signaling, which mediates growth, stress responses, and pathogenicity in fungi. Based on the pharmacological inhibition of calcineurin in *F. fujikuroi*, we discovered that calcineurin inhibitor FK506 or cyclosporin A can intensely prevent the growth of *F. fujikuroi*, and further investigated the feasibility of silencing calcineurin genes of *F. fujikuroi* in rice using host-induced gene silencing (HIGS) to confer resistance to bakanae disease. The constructs *FfCNA1-Ri* and *FfCNB1-Ri* were introduced into rice plants by using *Agrobacterium*-mediated gene transformation, and the copy numbers of transgenes were examined by Southern blot hybridization in the transgenic lines. The results of pathogen inoculation assay and fungal biomass quantification demonstrated that the transgenic rice plants that carry the *FfCNA1-Ri* or *FfCNB1-Ri* construct had increased resistance against bakanae disease. We propose that RNAi-derived siRNAs might efficiently suppress the expression of calcineurin genes in *F. fujikuroi*, leading to impaired growth and poor colonization of *F. fujikuroi* in rice. These findings indicate that HIGS might be a potential disease management strategy for rice bakanae disease.

## Introduction

Rice bakanae (also known as foolish seedling disease) caused by *Fusarium fujikuroi* [teleomorph: *Gibberella fujikuroi* (Sawada)] is widespread in the major rice-growing countries of Asia and Africa (Chen et al., 2016b; Sunani et al., 2019). As a disease that frequently occurs in rice-producing regions, the yield losses caused by the pathogen be up to 75%, posing a substantial threat to crop production (Matić et al., 2016; Piombo et al., 2020; Peng et al., 2022). The contaminated seeds primarily serve as the inoculum in the field. Furthermore, thick-walled hyphae and macroconidia can persist in plant debris and soil, leading to infections in the subsequent growing season (Carter et al., 2008; Chen et al., 2020; Tadasanahaller et al., 2023). Germinated conidia invade plants through seeds, stem bases, and root tips, subsequently establishing colonization within plant tissues (Sun, 1975). Under specific conditions, infected plants can produce a large quantity of conidia, which can be readily transmitted to nearby plants. Particularly during the heading stage, infected seeds initiate a new disease cycle in the subsequent season (Matic et al., 2017). During the seedling stage, infected rice plants show typical disease symptoms, such as spindly growth with thin and chlorotic leaves. As the disease progresses, *F*. *fujikuroi* colonies are aggregated in the vascular tissue and retard water transport, subsequently resulting in the severely infected seedlings dying at the early stages of grow or after transplanting into the field.

In crops, the predominant disease control strategy against *F. fujikuroi* is using fungicides to treat seeds. However, the increasing occurrence of the disease and developing resistance to fungicide in the fungal population have become a dilemma for disease management (Chen et al., 2016a; Niehaus et al., 2017). Thus, developing strategies providing credible, sustainable resistance to bakanae disease is necessary in crops, among which generation of *F. fujikuroi*-resistant cultivars might serve as one option.

An alternative approach to using fungicides involves RNA interference (RNAi), and is known as host-induced gene silencing (HIGS). RNAi has become a powerful tool for the characterization of gene function or generating disease resistant plants (Ghag, 2017). The RNAi processes are initiated with an RNase III enzyme (Dicer), which cuts the precursor double-stranded RNA (dsRNA) into double-stranded small interfering RNAs (siRNAs) of approximately 21 nucleotides (nt). These siRNAs are unwound into single-stranded siRNA by helicase, and the antisense siRNAs are then bound with several proteins, resulting in the formation of an RNA-induced silencing complex (RISC). The RISC is then base-paired with the endogenous complementary mRNA, leading to the cleavage of targeted mRNAs or blocking protein translation (Majumdar et al., 2017).

HIGS is the ectopic expression of siRNAs in transgenic plants in order to silence specific genes of pathogens or pests. HIGS generates siRNA molecules in plants targeting the specific mRNAs of pathogens and cause their degradation. Recently, the application of HIGS has been shown to be a powerful strategy for plants against phytopathogenic fungi and other pathogens (Nunes and Dean, 2012; Qi et al., 2018). During the pathogen-host interaction, the siRNA derived from transgenic plants can traffic into pathogens through an unidentified mechanism, which might result in targeted gene silencing, known as cross-kingdom RNA interference (ckRNAi) (Wang et al., 2016). The translocation mechanism of dsRNA/siRNA between the pathogen and the host relies on either a naked form or encapsulation within extracellular vesicles (EVs) (Wang and Dean, 2020; He et al., 2021; Mahanty et al., 2023). As a result, the efficiency of ckRNAi is positively correlated with the uptake of dsRNAs by fungi from the external environment. HIGS was used in wheat against *Fusarium* head blight with a specific chitin synthase gene from *Fusarium graminearum* constructed using RNAi and ectopic expression in transgenic wheat, and it was found that the chitin synthase gene was downregulated in *F. graminearum* and thereby conferred durable resistance to wheat head blight (Cheng et al., 2015). The dsRNA-derived siRNAs expressed in banana could silence two vital fungal genes *VEL* and *FTF1* in *Fusarium oxysporum* f. sp. *cubense* (Ghag et al., 2014), revealing that HIGS might be applicable for resistance against banana Fusarium wilt. The cyclic adenosine monophosphate protein kinase A (PKA) has been demonstrated to show vital roles in controlling pathogenicity, morphogenesis and development in phytopathogenic fungi (Fuller and Rhodes, 2012). The transgenic wheat plants expressing siRNAs targeting the *CPK1* (a PKA) gene of *Puccinia striiformis* f. sp. *tritici* (*Pst*) increased the resistance of wheat to stripe rust (Qi et al., 2018). The transgenic rice capable of expressing a specific dsRNA sequence was developed and evaluated for its effectiveness in combating *Rhizoctonia solani* AG-1 IA. The specific dsRNA sequence targets *AGLIP1*, an effector in the pathogen that induces cell death in rice protoplasts. The transgenic plant exhibited stable dsRNA expression and enhanced resistance against the pathogen, as evidenced by reduced infection areas and lower fungal biomass in the infected transgenic rice line (Mei et al., 2024). These studies provide important insight for developing stable transgenic plants using a HIGS-mediated silencing of specific genes in fungi.

Calcineurin is a calcium/calmodulin-dependent protein phosphatase and forms a heterodimer including a catalytic subunit A (Cna) and regulatory subunit B (Cnb) (Juvvadi et al., 2014). In the fungal kingdom, calcineurin maintains cellular processes as diverse as growth, morphogenesis, ion homeostasis, stress response, and virulence by triggering downstream targets (Hemenway and Heitman, 1999; Park et al., 2019). When fungal cells come across external stress, the cell compartment Ca^2+^ influx system is initiated and causes an augmented intracellular Ca^2+^ concentration. In the calcineurin signaling, calmodulin (CaM) and calcineurin Cnb subunit play as a sensor for the Ca^2+^ signal, then CaM/Ca^2+^ specifically binds to Cna and Cnb subunits of calcineurin to form a CaM/Ca^2+^-calcineurin complex, resulting in the activation of protein phosphatase activity. The activated calcineurin then dephosphorylates the downstream targets, such as Crz1, permitting their nuclear import and inducing the expression of the target genes (Juvvadi and Steinbach, 2015; Liu et al., 2015).

For phytopathogenic fungi, calcineurin also acts crucial role in pathogenicity. The previous studies had shown that calcineurin signaling pathway involves in multiple cellular processes (Chen et al., 2010; Juvvadi et al., 2014; Hou et al., 2020; Yadav and Heitman, 2023). Induction of antisense calcineurin expression in *Sclerotinia sclerotiorum* impaired sclerotial development at the prematuration phase. It resulted in the decrease of cell wall β-1,3-glucan content and attenuated pathogenesis in tomato and *Arabidopsis* (Harel et al., 2006). In *Magnaporthe grisea*, inhibition of calcineurin by cyclosporin A (CsA) was found to inhibit hyphal growth, appressorium development and retarded infection structure formation, indicating that calcineurin is vital for the formation of infection structure in *M. grisea* (Viaud et al., 2002, Viaud et al., 2003). In *Fusarium graminearum*, the calcium-calcineurin pathway involved in fungicide sensitivity. Either by chelating Ca^2+^ or inhibiting calcineurin with CsA, the inhibitory effect of tebuconazole was enhanced (Wang et al., 2023). Moreover, the Cna subunit of calcineurin was required for the morphogenesis and ear gall formation in *Ustilago maydis* (Egan et al., 2009). However, the roles of calcineurin in growth and pathogenicity have not yet been studied in *F. fujikuroi*.

In this study, we demonstrate the feasibility of HIGS-based silencing of calcineurin genes of *F. fujikuroi* in rice to confer resistance to bakanae disease. In bioassays, transgenic rice that carried the *FfCNA1*-RNAi or *FfCNB1*-RNAi construct showed a high level of resistance toward *F. fujikuroi* infection. Our results showed that RNAi-derived siRNAs might efficiently suppress the expression of calcineurin genes in *F. fujikuroi*, leading to impaired growth and poor colonization of *F. fujikuroi* in rice. These results indicate that HIGS might be a potential disease management strategy for rice bakanae disease.

## Results

### Identification of Cna1 and Cnb1 in *Fusarium fujikuroi*

**Figure 1:**
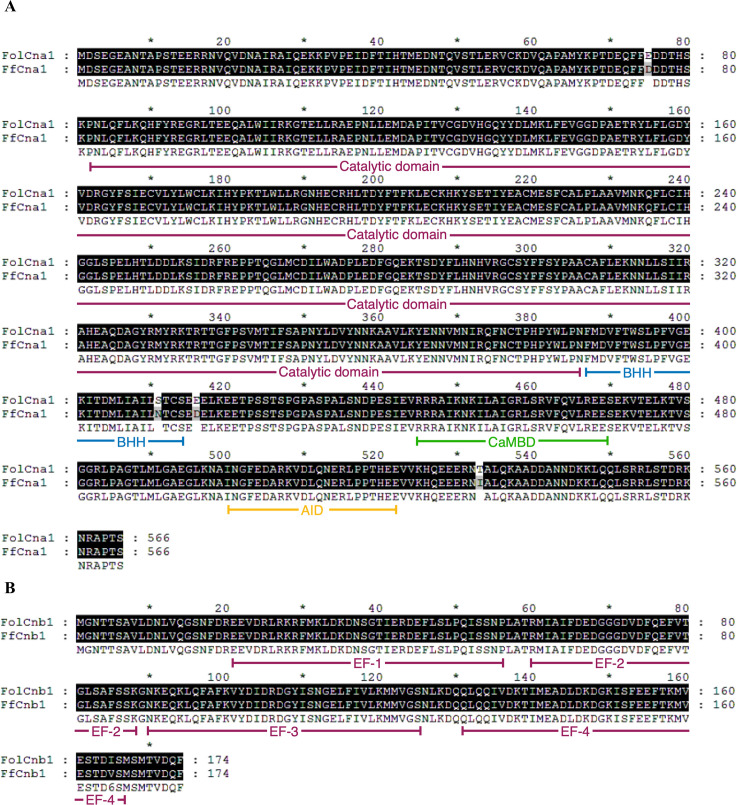
Domains and motifs of *Fusarium fujikiuroi* calcineurin catalytic (Cna1) and regulatory (Cnb1) subunits. Amino acid alignment of FfCna1 **(A)** and FfCnb1 **(B)** with homologs from *F*. *oxysporum* f. sp. *lycopersici*. Identical amino acids are shown by the white letters on the black background. The results indicate that FfCna1 contains a catalytic domain, a Cnb1 binding helix (BBH), a CaM-binding domain (CaMBD) and an autoinhibitory domain (AID), while FfCnb1 contains four Ca^2+^-binding motifs (EF).

**Figure 2:**
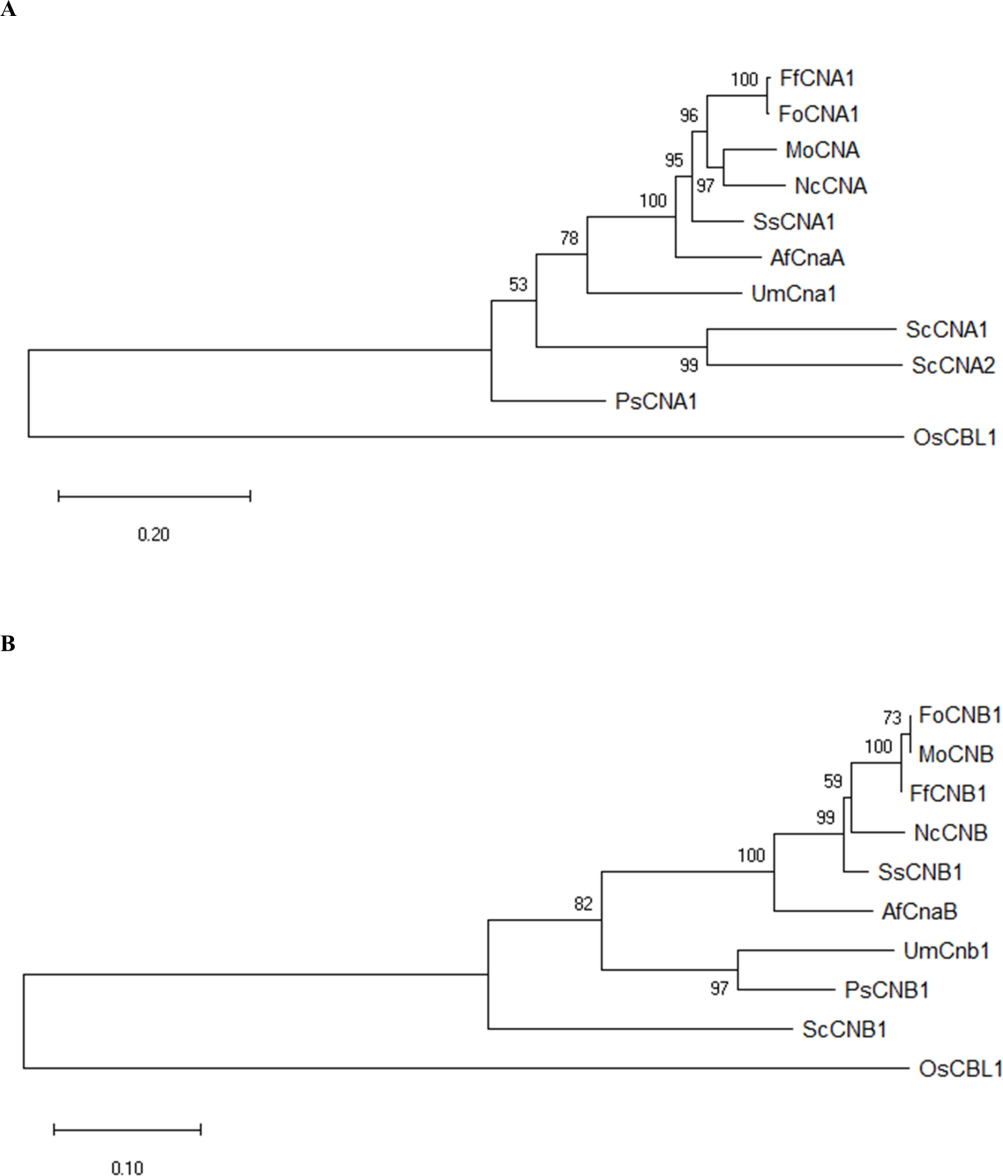
Phylogenetic analysis of *F*. *fujikuroi* CNA1 and CNB1 subunits. Phylogenetic tree of FfCNA1 **(A)** and FfCNB1 **(B)** with homologs from model organisms and phytopathogens. In addition, the calcineurin-B like protein of rice was also included. The phylogenetic tree was constructed in MEGA software using the neighbor-joining method. Confidence of groupings was estimated by using 1,000 bootstraps. GenBank accession number of proteins: FfCNA1 and FfCNB1 (*Fusarium fujikuroi*, XP_023429196 and XP_023428777), FoCNA1 and FoCNB1 (*Fusarium oxysporum* f. sp. *lycopersici* 4287, XP_018243626 and XP_018234243), MoCNA and MoCNB (*Magnaporthe oryzae* 70-15, XP_003711354 and XP_003709672), NcCNA and NcCNB (*Neurospora crassa* OR74A, XP_961193 and CAA73345), SsCNA1 and SsCNB1 (*Sclerotinia sclerotiorum* 1980 UF-70, XP_001597594 and XP_001598128), AfCnaA and AfCnaB (*Aspergillus fumigatus* Af293, XP_753703 and XP_747624), UmCna1 and UmCnb1 (*Ustilago maydis*, AAP48999 and EAK82139), ScCNA1 and ScCNA2 (*Saccharomyces cerevisiae*, NP_013537 and NP_013655), ScCNB1 (*Saccharomyces cerevisiae*, NP_012731), PsCNA1 and PsCNB1 (*Puccinia striiformis* ***f***. sp. *tritici*, AFW98882 and AFW98883), OsCBL1 *(Oryza sativa* Japonica Group, NP_001391328).

To identify genes encoding calcineurin catalytic subunit Cna1 and regulatory subunit Cnb1 protein in *F. fujikuroi*, a BLAST search was conducted in NCBI (https://www.ncbi.nlm.nih.gov/) by using the amino acid sequences of Cna1 (XP_018243626) and Cnb1 (XP_018234243) in *F. oxysporum* f. sp. *lycopersici*. BLAST results revealed FoCna1 and FoCnb1 orthologs in *F. fujikuroi* (*Ff*) designated as FfCna1 (XP_023429196) and FfCnb1 (XP_023428777), respectively. The predicted *FfCNA1* has an open reading frame (ORF) of 1698 bp, encoding a protein with 565 amino acids, which contains a catalytic domain, an autoinhibitory domain, a Cnb1 binding helix, and a CaM-binding domain (**Figure 1A**). The *FfCNB1* has an ORF of 522 bp that encodes a protein of 173 amino acids, which contains four Ca^2+^-binding motifs (**Figure 1B**). Sequence alignments of amino acid showed that FfCna1 shares 99%, 87%, and 86% identity with the calcineurin A subunit of *F. oxysporum* f. sp. *lycopersici*, *Sclerotinia sclerotiorum*, and *Magnaporth oryzae*, respectively. The FfCnb1 shared 99% identity with the calcineurin B subunit in *F. oxysporum* f. sp. *lycopersici*, and 94% to both *S*. *sclerotiorum* and *M*. *oryzae*. The phylogenetic trees revealed that *F. fujikuroi* calcineurin was closely related to the calcineurin of *F. oxysporum* f. sp. *lycopersici* but was distant to those in yeasts (**Figure 2**).

### *In vitro* effect of calcineurin inhibitors on the growth of *F. fujikuroi*

**Figure 3:**
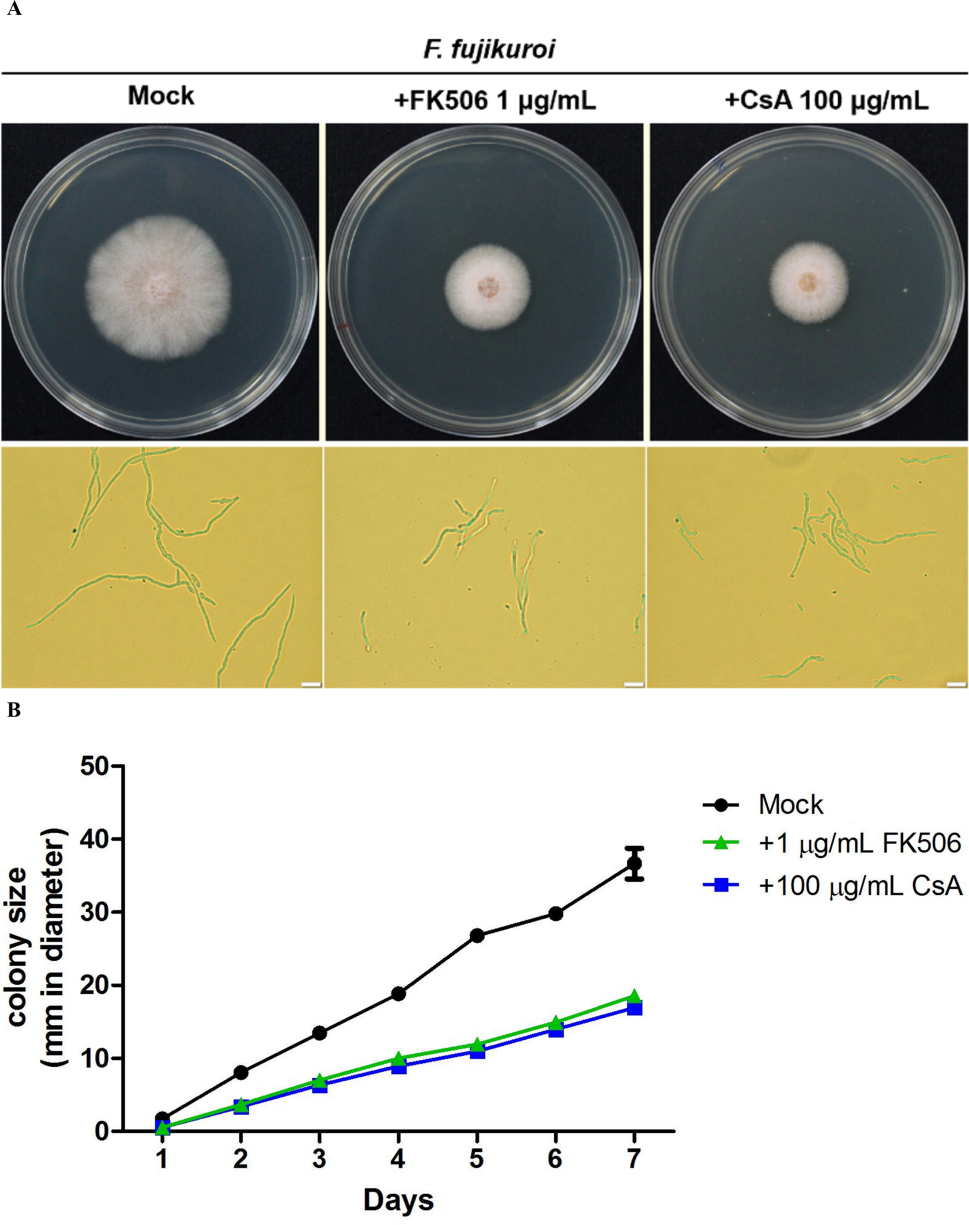
Pharmacological inhibition of *F*. *fujikuroi* calcineurin by calcineurin inhibitors. **(A)** Phenotypes of the *F*. *fujikuroi* IL01 in the presence or absence of a calcineurin inhibitor. The mycelia agar discs of *F*. *fujiluroi* IL01 were inoculated on plates containing either FK506 (1 μg/mL) or cyclosporin A (CsA, 100 μg/mL) and incubated at 25°C for 7 days. Bar, 20 μm. **(B)** Growth kinetics of *F*. *fujikuroi* were obtained from **(A)**. The experiments were performed in triplicate. Statistical significance was analyzed by ANOVA, compared to mock and calcineurin inhibitor treated *F*. *fujikuroi* (*P*< 0.001). Error bars represented standard deviations.

Calcineurin inhibitors were used to examine the roles of calcineurin on the growth of *F. fujikuroi*. The mycelia agar discs of *F. fujikuroi* IL01 were inoculated on PDA medium in the absence or presence of FK506 (1 mg/mL) or cyclosporin A (CsA, 100 mg/mL), and incubated at 25°C for 7 days. The average diameter of the *F. fujikuroi* mycelia colony was able to extend 36.3 ± 2.1 mm in the inhibitor-free medium after 7 days of incubation (**Figure 3** **).** Whereas in the medium containing FK506 or CsA, the average diameter of the colonies was only 18.5 ± 0.2 and 16.9 ± 0.1 (*P<* 0.001). These results indicated that calcineurin is critical for *F. fujikuroi* growth.

### Generation of RNAi constructs and transgenic rice

**Figure 4:**
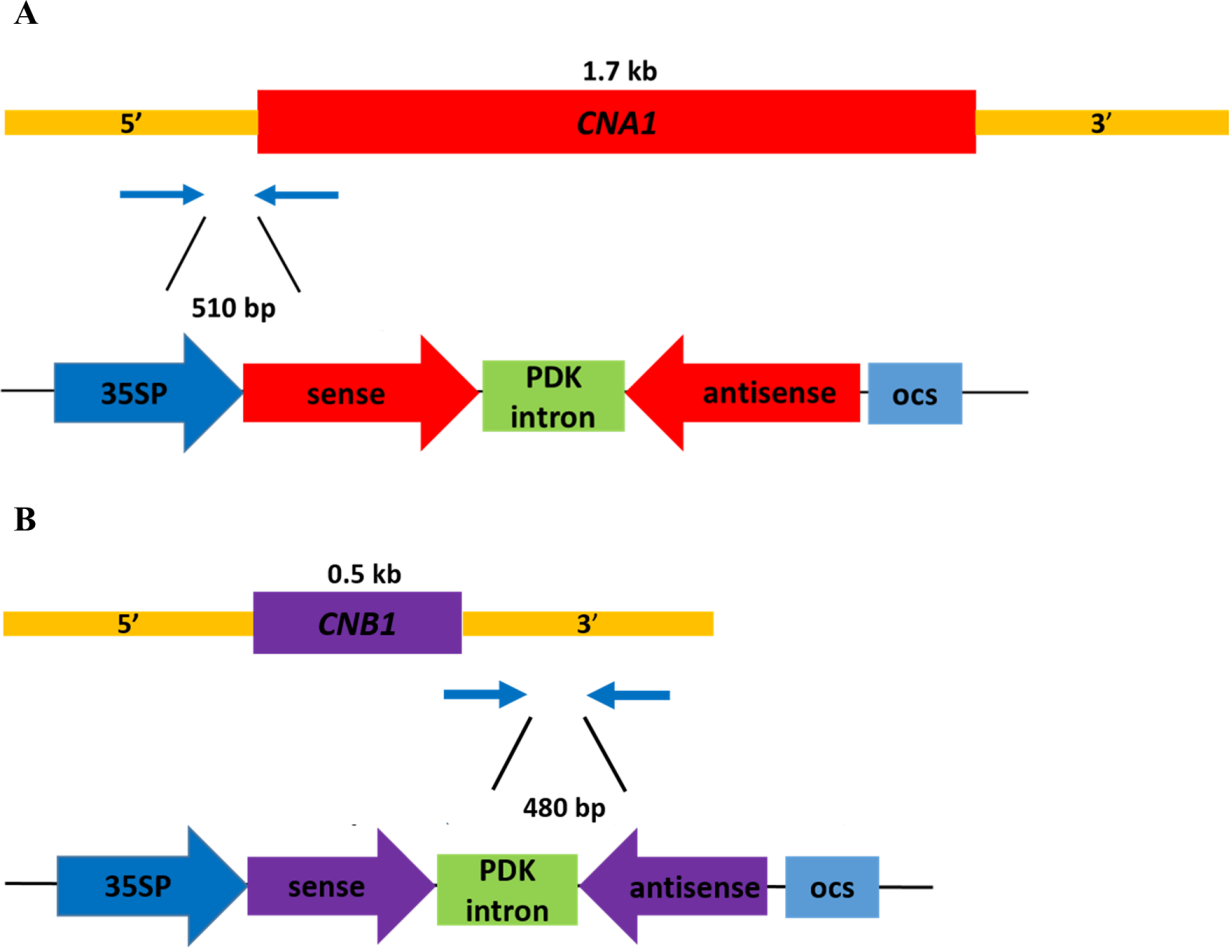
Schematic diagram of the RNA interference expression construct of calcineurin genes of *F*. *fujikuroi* in transgenic rice for HIGS analysis. The 510 bp DNA fragment containing the 5’-untranslated region and the 5’-coding sequence of *F*. *fujikuroi CNA1* **(A)**, and a 480 bp DNA fragment containing 3’-coding sequence and 3’-untranslated region of *F*. *fujikuroi CNB1* **(B)** were amplified by PCR, and inserted downstream of the 35S promoter in the sense and antisense orientation, with the PDK intron as the junction. 35SP, CaMV 35S promoter; PDK intron, pyruvate dehydrogenase kinase intron; ocs, octopine synthase terminator.

To investigate whether HIGS can be applied to combat rice bakanae disease, the RNAi strategy was performed for specific targeting of *F. fujikuroi* calcineurin mRNA transcripts. For the *FfCNA1*-RNAi construct, a 503 bp fragment of *FfCNA1* gene containing the 5’-untranslated region (328 bp) and 5’-coding sequence (175 bp) was PCR amplified from *F. fujikuroi* genomic DNA using primer sets JC1440/JC1441 and JC1442/JC1443. The 503 bp DNA fragment was fused up and downstream of the PDK intron in the vector pKANNIBAL (Helliwell and Waterhouse, 2003), and the inverted repeat of *FfCNA1* construct was expressed under the control of the CaMV 35S promoter (**Figure 4A**) to generate the *FfCNA1-Ri* construct in transgenic rice. Likewise, a 439 bp DNA fragment of *FfCNB1* containing the 3’-coding region (11 bp) followed by the 3’-untranslated region (428 bp) was amplified with primer pairs of JC1444/JC1445 and JC1446/JC1447. The construct of *FfCNB1-Ri* (**Figure 4B**) was similar to the *FfCNA1-Ri* described above. The *FfCNA1-Ri* and *FfCNB1-Ri* constructs were then cloned into the pBH binary vector (Ho et al., 2000) (**Figure 4**). The rice calli induced from the immature embryo were used for the *Agrobacterium*-mediated gene transformation. Under hygromycin B selection, 7 and 11 T0 independent antibiotic resistant transgenic lines of *FfCNA1-Ri* and *FfCNB1-Ri* were obtained, respectively, and used for further studies.

### Molecular analysis of transformants

**Figure 5:**
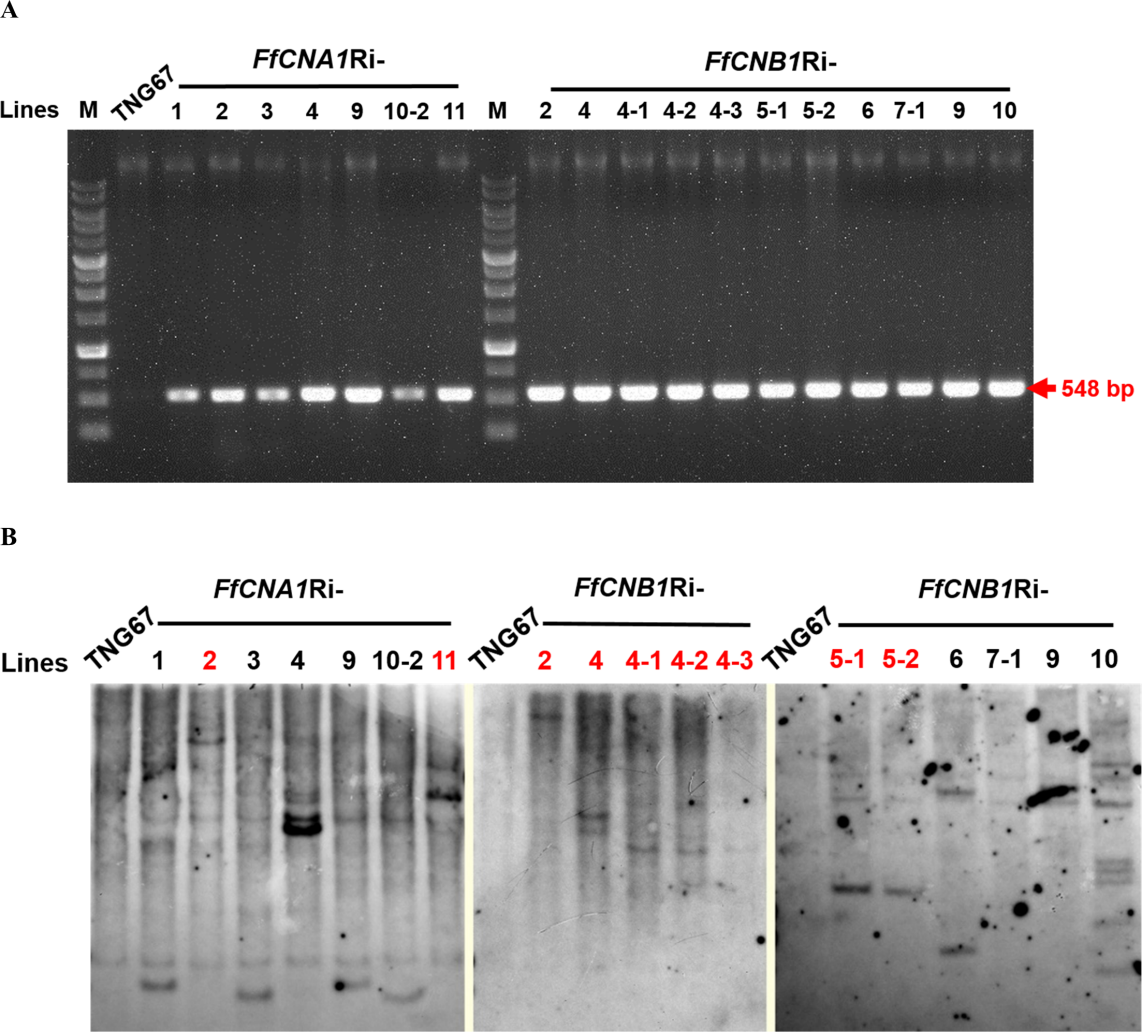
PCR and Southern blot analysis of rice wild-type (TNG67) and transgenic lines. Genomic DNAs were purified from the calli of TNG67, *FfCNA1*Ri, and *FfCNB1*Ri transgenic lines, respectively. **(A)** PCR analysis using the *FfCNA1-Ri* and *FfCNB1-Ri* specific primer sets. **(B)** Genomic DNAs were digested by *Pst*I and subjected to agarose gel electrophoresis, followed by Southern blot hybridization using the alkaline phosphatase-labeled hygromycin phosphotransferase (*Hph*) gene as the probe. The single-copied transgenic lines are marked with red letters.

To confirm whether the hygromycin B resistant lines contained the transgenes, the antibiotic selection marker *hygromycin phosphotransferase gene* (*Hph*) contained in the transgene cassette was amplified by PCR. The genomic DNA was purified from the T1 seedlings of the putative transgenic lines, and then used as template for PCR amplification As shown in **Figure 5A**, the results revealed that all the putative transgenic lines could produce the targeted DNA fragments (548 bp), whereas there was no signal detected in the wild-type control. These results demonstrated that all putative transgenic plants (*FfCNA1-Ri* and *FfCNB1-Ri* lines) contained the targeted transgenes. Moreover, to prevent the segregation and recombination of multiple copies of transgenes in the next generation of offspring, and to verify the transgenic lines that are consistent with the expression levels of transgenes, it is necessary to screen the transgenic lines carrying single copy of transgene. Southern blot analysis was performed to select single-copy transgenic lines, ensuring stable integration of the transgenes in the genome of transgenic rice plants. Because the T-DNA in the binary vector has only one restriction site *Pst*I, the number of hybridization signals detected on the x-ray films after chemiluminescence corresponded to the copy number of the T-DNAs integrated into the rice genome in these transgenic lines. After hybridization with an alkaline phosphatase-labeled *Hph* probe, in *FfCNA1-Ri* transgenic plants, we found that two independent lines, *FfCNA1-Ri*-2 and *FfCNA1-Ri*-11, exhibited a single copy of the transgene. In *FfCNB1**-**Ri* transgenic plants, we identified that three independent lines, *FfCNB1-Ri-2*, *FfCNB1-Ri-4* and *FfCNB1-Ri-5*, had one copy of the transgene (**Figure 5B**). All single-copy transgenic rice plants were healthy and showed a growth phenotype similar to the wild-type plants.

### *FfCNA1-Ri* and *FfCNB1-Ri* transgenic rice plants exhibit enhanced resistance to *F. fujikuroi* infection

**Figure 6:**
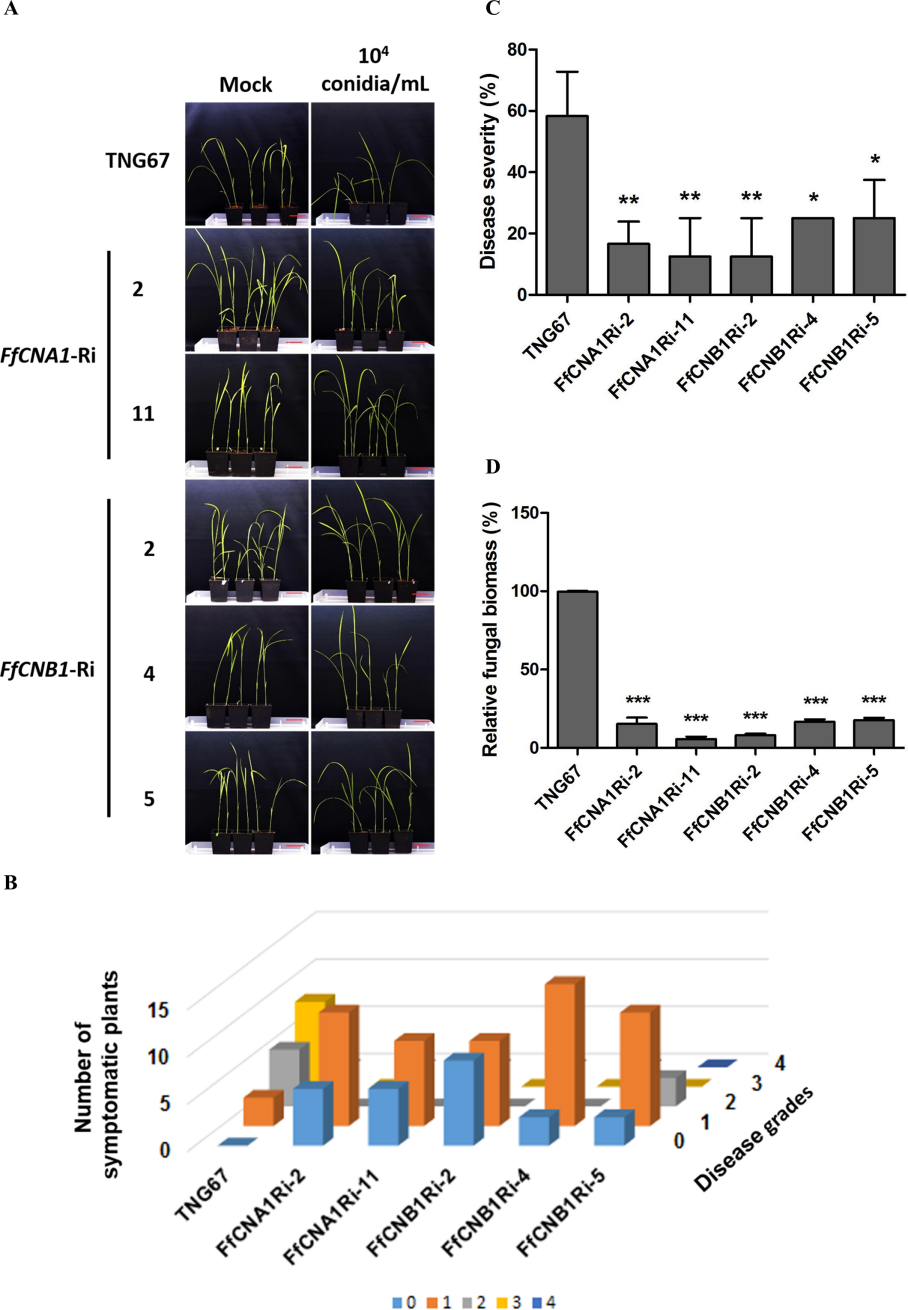
Ectopic expression of *FfCNA1-Ri* and *FfCNB1-Ri* in transgenic rice plants conferred enhanced resistance against rice bakanae disease. **(A)** The phenotypes of wild-type and transgenic rice lines at 21 dpi. Bar, 5 cm. The three days of germinated rice seeds were inoculated with *F*. *fujikuroi* (10^4^ conidia/mL) for 1 h and incubated in a growth chamber at 28°C for 21 days. The disease index **(B)** and the disease severity **(C)** levels were calculated at 21 dpi based on criteria described in materials and methods. **(D)** The fungal biomass was determined by qPCR in rice seedlings at 21 dpi. Error bars represent standard deviations, and the relative fungal biomass was estimated by the expression levels of *F*. *fujikuroi TEF1-α*, which were normalized to the rice *Actin* gene expression level. These results were obtained from three biological replicates. Asterisks indicated significant differences compared to wild-type according to ANOVA. *, *P*< 0.05; **, *P*< 0.01; ***, *P*< 0.001.

To study the effects of *F. fujikuroi* IL01 on rice seedlings, a disease model of IL01 infected rice seedlings was established. Results showed typical bakanae symptoms in wild-type rice infected with *F. fujikuroi* IL01, such as internode elongation, large leaf angle, and seedling slender (**Figure 6A**). A pathogen infection assay was further conducted to evaluate the resistance toward *F. fujikuroi* between wild-type and transgenic rice seedlings. The T2 transgenic rice seeds derived from all the single-copy transgenic lines and the wild-type were dehulled and sterilized with sodium hypochlorite solution, and germinated on 1/2 MS medium with (transgenic lines) or without (wild-type) hygromycin B to remove the revertant seeds (wild-type). After culturing for 4 days, most of the wild-type and transgenic seeds had germinated, whereas the embryos from the revertant seeds became black. The seeds that had been germinated for 4 days were inoculated with *F. fujikuroi* (10^5^ conidia/mL) and grown at 28°C. To determine the levels of disease severity on pathogen-infected rice seedlings, the disease severity index (DSI) was divided into 0–4 levels, and disease severity was calculated at 21 days post-infection(dpi). The disease severity (%) in wild-type was 58.3 ± 8.3% (DSI3), whereas in *FfCNA1-Ri*-2, -11 and, *FfCNB1-Ri*-2, -4, and -5 were 16.6±4.1% (DSI1), 12.5 ± 7.2% (DSI1), 12. 5 ± 7.2% (DSI1), 25.0 ± 0.0% (DSI1), and 25.0 ± 7.2% (DSI2), respectively (**Figures 6B, C**), that were significantly, 71.5%, 78.6%, 78.6%, 57.1% and 57.1% reduced in severity compared with that of the wild-type seedlings (*P<* 0.01 in *FfCNA1-Ri*-2, -11 and, *FfCNB1-Ri*-2; *P<* 0.05 in *FfCNB1-Ri*-4, and -5). Moreover, infected wild-type seedlings were wilted and died at 30 dpi, whereas all the *FfCNA1-Ri* and *FfCNB1-Ri* plants dramatically reduced disease symptoms and no dead plants were observed.

### Quantification of *F. fujikuroi* biomass in transgenic rice plants

To further assess the effect of siRNA-mediated HIGS on pathogen-infected rice, real-time quantitative PCR (qPCR) was performed to quantify the fungal biomass in transgenic lines and the wild-type plant. After 21 dpi, the genomic DNA was purified from the *F. fujikuroi*-infected wild-type, *FfCNA1*-*Ri*, or *FfCNB1-Ri* seedlings. The qPCR was performed by using the species-specific region of translation elongation factor 1-α (*TEF1-α*) gene in *F. fujikuroi*. The relative amount of *TEF1-α* was normalized with the rice *Actin 1* gene, and these values represent the biomass of *F. fujikuroi* in infected rice. As shown in **Figure 6D**, the relative biomass of *F. fujikuroi* in the wild-type seedling was 99.6 ± 60.3%. Whereas in the *FfCNA1-Ri*-2 and -11 and, the *FfCNB1-Ri*-2, -4 and -5 were 15.3 ± 2.3%, 5.6 ± 0.8%, 8 ± 0.5%, 16.6 ± 0.8%, and 17.6 ± 0.8%, respectively, that were significantly 84.7%, 94.4%, 91.9%, 83.4% and 82.4% reduced compared to that of the wild-type seedling (*P<* 0.001). These results revealed that expression of *FfCNA1*-*Ri* or *FfCNB1-Ri* in transgenic rice could enhance resistance against *F. fujikuroi* infection. Out of these, the transgenic lines *FfCNA1-Ri*-11 and *FfCNB1-Ri*-2 demonstrated higher disease resistance than other transgenic lines.

## Discussion

Recently, HIGS has been used as an innovative approach for managing plant diseases. Numerous studies have shown the successful application of HIGS, and that genes crucial for morphogenesis or pathogenesis are the ideal targets for silencing (Nunes and Dean, 2012; Ghag, 2017). Calcineurin is well known for regulating hyphal growth, septation, and virulence in many plant and human pathogenic fungi. Moreover, calcineurin has been proven to be involved in the development of propagule or structure, which is required for infection, such as asexual/sexual spores, sclerotium, and appressorium (Chen et al., 2010; Park et al., 2019). Furthermore, our previous research showed that calcineurin plays an important role in the growth and virulence of *Fusarium oxysporum* f. sp. *lycopersici* (Hou et al., 2020). According to the pharmacological inhibition of calcineurin, our study revealed that both FK506 and CsA could lead to blunted and irregularly branched hyphae in *F. fujikuroi*, suggesting that calcineurin might be involved in hyphal development and further resulted in the defect of pathogenesis. To further evaluate the efficiency of dsRNA in RNAi-mediated gene silencing, naked dsRNA was applied to *F. fujikuroi* colony. The observed differences in colony growth under different dsRNA treatments suggest that the fungal growth was inhibited by exogenous dsRNA (**Supplementary Figure S1**). As a result, *F. fujikuroi* calcineurin could be the potential target for disease management via an RNAi approach.

Bioinformatic analysis and phenotypic assay were performed to assess the probability of off-target effects as the calcineurin cascade is conserved in eukaryotes and involved in many biological processes. To this end, 5’ or 3’ untranslated regions of *FfCNA1* and *FfCNB1* were chosen to build the RNAi constructs for the HIGS approach. According to phylogenetic analyses, results showed that rice calcineurin B-like protein (OsCBL1) was located at the most distance from *F. fujikuroi* and other fungal calcineurins. Furthermore, sequence alignment determined that our RNAi target sequences did not have any remarkable homology with the rice genome, and there was no difference in growth phenotype observed between the wild-type and transgenic rice lines (**Figure 6A**). Moreover, the expression of *OsCBL7*, which exhibited the highest sequence identity towards *FfCNB1* among rice calcineurin B-like protein family, showed no significant difference between wild-type and transgenic rice (**Supplementary Figure S2**). Taken together, these findings indicate that siRNA production does not influence the expression of specific genes or the physical state of rice. The off-target effect toward important genes in arbuscular mycorrhizal fungi (AMF) or plant growth-promoting rhizobacteria (PGPR) present in rice roots were also considered. The AMF species mostly belong to the *Glomeraceae* family, including *Rhizophagus* spp.*, Glomus* spp. and *Funneliformis* spp. (Vallino et al., 2014; Wang et al., 2015). The results of alignment revealed poor similarity between the nucleotide sequence of calcineurin subunit A and B in *R. irregularis* compared with sequences used in *FfCNA1-Ri* and *FfCNB1-Ri* construct (**Supplementary Figure S3**). In contrast to eukaryotes, bacteria lack direct RNAi machinery, but instead use DNA as a gene silencing signal for the initiation of the CRISPR/Cas system, thus, it is unlikely that siRNA can affect the growth of PGPR. Moreover, the specificity of dsRNAs was evaluated. The results indicated that the presence of dsRNA has modestly impact on the virulence of *F. fujikuroi* when the dsRNA sequence was not homologous to the *F. fujikuroi* genome (**Supplementary Figure S4**). Taken together, siRNAs derived from the *FfCNA1*- or *FfCNB1-Ri* constructs might reduce the off-target effect on rice plants or nearby organisms.

As shown in **Figure 6**, the disease severity and the relative fungal biomass were lower in transgenic rice seedlings than in the wild-type. In addition to assessing disease severity, siRNA detection was conducted in transgenic rice to determine whether the observed enhancement in resistance was mediated by siRNAs derived from the sequences. Sequence alignment identified siRNAs ranging from 18 to 30 nucleotides in length that were complementary to the target sequence in *FfCNB1-Ri-4*, whereas no corresponding siRNAs were detected in WT (**Supplementary Figure S5**). These results indicated the expression of RNAi transgenes (*FfCNA1*-*Ris and FfCNB1-Ris*) in rice for the formation of anti-calcineurin siRNA induced the effect of HIGS to improve disease resistance in rice. Recently, HIGS in plants has been reported to combat diseases caused by *Fusarium* species. For instance, expression of the *F. graminearum* essential gene *Chs3b* (chitin synthase) using RNAi in wheat could increase resistance to Fusarium head blight (Cheng et al., 2015). Ectopic expression of fungal CYP51 (cytochrome P450 lanosterol C-14α-demethylase) RNAi construct in Arabidopsis and barley was able to enhance resistance against *F. graminearum* (Koch et al., 2013). Resistance against banana Fusarium wilt was also achieved by using the HIGS strategy, via targeting the conserved domain of the fungal morphogenesis related genes, the velvet protein family, resulting in growth inhibition and reduced disease severity (Ghag et al., 2014). These studies also emphasized that genes which play a critical role in growth and virulence of pathogens can act as ideal HIGS targets to protect plants from pathogen attack (Qi et al., 2019).

In this study, by using a HIGS strategy in rice to directly silence *FfCNA1* and *FfCNB1* in *F. fujikuroi*, we found that the transgenic rice seedlings had enhanced resistance against *F. fujikuroi* infection, indicating calcineurin is critical for *F. fujikuroi* to infect rice plants. Furthermore, our results also provide insights into the mechanism of HIGS in plants and a strategy for improving resistance against rice bakanae disease.

## Materials and methods

### Fungal strain and culture condition

The *Fusarium fujikuroi* strain IL01 was used in this study (Hsu et al., 2013). *F. fujikuroi* was grown on potato dextrose agar (PDA; 0.4% potato starch from infusion, 2% dextrose, 1.5% agar) (BioShop, Burlington, ON, Canada). The growing temperature of *F. fujikuroi* was set at 25°C.

### Colony morphology observation

*F. fujikuroi* was streaked out from -80°C stock, incubated on YPD at 25°C for one week, and cut to 7 mm in diameter with a hole punch (Bioshop, Canada). Agar discs having cultures were put on PDA plates, PDA with 1 μg/mL FK506 (Astellas Pharma, Tokyo, Japan) or 100 μg/mL cyclosporin A (LC Laboratories, Woburn, USA) with three replicates. All plates were incubated at 25°C for seven days, and the diameter of the fungal colony was measured daily. Data were analyzed by two-way ANOVA and Bonferroni post-test by using GraphPad Prism 5 (GraphPad Software, CA, USA). The representative plates were photographed.

### Plant DNA isolation and Southern blotting

Rice genomic DNA was extracted from calli or young leaves. Tissues were frozen in liquid nitrogen and ground into powder. The powder was transferred to a 1.5 mL microcentrifuge tube followed by adding equal volume of urea extraction buffer [8M urea, 0.35M NaCl, 0.05M Tris-HCl (pH 7.5), 0.02M EDTA, 2% SDS] and a 3:2 ratio mixture of phenol:chloroform, mixed well and then centrifuged at 14,000 rpm for 10 min at 4°C Afterward, the upper phase was collected and an equal volume of isopropanol was added to precipitate the nucleic acid. The extracts were centrifuged at 14,000 rpm for 5 min. The supernatants were thrown away and the nucleic acid pellets were washed in 70% ethanol followed by 100% ethanol. The pellets were air dried for 5 min and dissolved with sterile water.

For Southern blot analyses of transgenic rice plants, 50 µg DNA was digested with *Pst*I and separated in a 0.8% agarose gel at 20V for 10 h. After electrophoresis, the gel was depurinated in 0.2N HCl for 10 min followed by soaking in denaturation solution (0.5 M NaOH, 1.5 M NaCl) and neutralization solution [0.5 M Tris-HCl (pH 8.0), 1.5 M NaCl] for 2 h, respectively. The hygromycin phosphotransferase (*Hph*) DNA probe was labeled with alkaline phosphatase by using Amersham AlkPhos Direct Labeling and Detection Systems (GE Healthcare, USA). The DNA fragments were transferred to a Hybond-N^+^ membrane (GE Healthcare, USA). The primers used for the DNA probe generated by PCR are shown in **Table 1**.

**Table 1:** Primers used in this study.

Primer	Use	Sequence (5’ to 3’)
JC1440	F-*FfCNA1-Ri*-forward	ATACTCGAGCAGTGGCCCTTAGGTTCCTG
JC1441	R-*FfCNA1-Ri*-forward	ATAGGTACCCTTTGCACACACGCTCCAAG
JC1442	F-*FfCNA1-Ri*-reverse	ATATCTAGACAGTGGCCCTTAGGTTCCTG
JC1443	R-*FfCNA1-Ri*-reverse	ATCAAGCTTCTTTGCACACACGCTCCAAG
JC1444	F-*FfCNB1-Ri*-forward	ATACTCGAGACCAGTTCTAAACGATCGC
JC1445	R-*FfCNB1-Ri*-forward	ATAGGTACCAGACAGAATCAAGATGTTCG
JC1446	F-*FfCNB1-Ri*-reverse	ATATCTAGAACCAGTTCTAAACGATCGC
JC1447	R-*FfCNB1-Ri*-reverse	ATAGGATCCAGACAGAATCAAGATGTTCG
JC1676	F-*Hph* probe	TATGTTTATCGGCACTTTGC
JC1677	R-*Hph* probe	TGCTCCATACAAGCCAACCACG
JC1452	F-*FoAct* for qPCR	ATCCACGTCACCACTTTCAA
JC1453	R-*FoAct* for qPCR	TGCTTGGAGATCCACATTTG
JC1808	F-*FfTEF1*-α for qPCR	ATCCTGACCAAGATCTGGCGGGGTATATCTCA
JC1809	R-*FfTEF1*-α for qPCR	GCTCAGCGGCTTCCTATTGTCGAATGTTTAGTTTG
JC1814	F-*OsAct1* for qPCR	CCAGGCCGTCCTCTCTCTGTAT
JC1815	R-*OsAct1* for qPCR	AATGAGTAACCACGCTCCGTCA

The underline represents a restriction enzyme cutting site.

### Plant infection assay

The rice cultivar *Oryza sativa* L. cv Tainung 67 (TNG67, wild-type) and the transgenic rice seedlings were inoculated with *F. fujikuroi*. Three-day-old germinated seeds were inoculated with a suspension of 10^5^ conidia/mL of *F. fujikuroi* for 1 h, and then planted in horticultural substrate [peat moss: Akadama soil (1:1)], and grown in the chamber at 28˚C (16 h light/8 h dark). Disease severity was recorded at 21 days-post-inoculation (dpi). Data were plotted using software GraphPad Prism 5 (GraphPad Software, CA, USA). The disease severity index (DSI) was graded into five degrees from 0 to 4: 0 = no symptoms; 1 = only one symptom: internode elongation, large leaf angle, seedling slender or pale; 2 = two symptoms as described in 1; 3 = complex symptoms; and 4 = plant either wilted or dead. The DSI was modified from previous studies (Amatulli et al., 2010; Hsu et al., 2013; Chen et al., 2015). Six plants were used for each treatment. Data were analyzed by one-way ANOVA and Dunnett’s post-test using GraphPad Prism 5. The formula of disease severity is shown below:


$$\begin{array}{c} Disease\;severity\\ =\frac{\sum \left(number\;of\;symptomatic\;plants\times disease\;grade\right)\;}{total\;number\;of\;disease\;scale\times maximum\;disease\;grade}\\ \times 100\% \end{array}$$

### Pathogen biomass quantification in planta

Rice seeds were inoculated with or without *F. fujikuroi* and incubated in a growth chamber at 28°C (16 h light/8 h dark). After 21 dpi, seedlings were harvested and whole plants were cut in pieces and ground into a powder with liquid nitrogen for genomic DNA isolation as described above. Real-time quantitative PCR (qPCR) was carried out using 30 ng of genomic DNA as template with SYBR^®^Green PCR Master Mix (Thermo Fisher Scientific, Vilnius, Lithuania) in a StepOnePlus machine (Applied Biosystems, Foster City, CA, USA). The species-specific region of *F. fujikuroi TEF1-α* gene (Chen et al., 2016b) was amplified for fungal biomass measurement, and the rice *Actin* gene was used as internal control for sample equilibration, by using the 2^-ΔΔCt^ method. The average fungal biomass was examined using three plants for each line, and data were plotted using software GraphPad Prism 5. The primer sets that were used for detecting *FfTEF1-α* and *OsActin* genes are listed in **Table 1**.

### Plant materials and callus induction

The rice cultivar TNG67 was used in this study. The immature seeds were de-hulled and sterilized with 2.4% NaOCl for 1 h, then washed with sterile water and cultured on N6 solid medium (pH 5.7) (Duchefa Biochemie, Netherlands) containing 2 mg/L of 2,4-dichlorophenoxyacetic (2,4-D) (N6D medium) and sucrose (30 g/L) for callus induction. One month after, the callus derived from scutellum was subcultured to fresh N6D medium plus sucrose (30 g/L) for 10 days for *Agrobacterium*-mediated gene transformation.

### Construction of the *FfCNA1-Ri* and *FfCNB1-Ri* expression vectors

To construct the *35S::FfCNA1-RNAi* expression vector, a 0.5-kb DNA fragment including the 5’-untranslated region (328 bp) and 5’ coding region (175 bp) of *FfCNA1* was PCR-amplified using the primer sets JC1440/JC1441 and JC1442/JC1443 (**Table 1**). Both ends of DNA fragments were cleaved either with *Xho*I and *Kpn*I, or with *Xba*I and *Hin*dIII, and introduced into the same cloning sites of the vector pKANNIBAL (Helliwell and Waterhouse, 2003) in an inverted repeat manner, respectively. To construct a vector for the ectopic expression of *35S::FfCNB1-RNAi* in rice, a 439-bp DNA fragment that included 3’ coding region (11 bp) and 3’-untranslated region (428 bp) of *FfCNB1* was amplified using primer sets JC1444/JC1445 and JC1446/JC1447 (**Table 1**). Both ends of DNA fragments were cleaved either with *Xho*I and *Kpn*I, or with *Xba*I and *Bam*HI, and inserted into the vector pKANNIBAL in an inverted repeat manner, respectively, under the governor of 35S promoter. Both *35S::FfCNA1-Ri* and *35S::FfCNB1-Ri* constructs were linearized by *Pst*I digestion and introduced into the *Pst*I site of the pBH binary vector, respectively (Ho et al., 2000), followed by *Agrobacterium*-mediated transformation.

### Rice transformation

The *FfCNA1-Ri* or *FfCNB1-Ri* vectors were transformed into *Agrobacterium tumefaciens* strain EHA101 through electroporation, and the rice calli were co-cultured with the transformed *Agrobacterium* strain in the solid N6D medium (pH 5.2) containing 100 μM acetosyringone for 3-5 days. The calli were then washed thoroughly with sterile water and thereafter incubated on N6D medium supplemented with hygromycin B (50 mg/L) and cefotaxime (250 mg/L) for 30 days. After incubation, the newly generated transformed rice cells (callus) were treated with osmotic stress by transferring to the N6 medium plus sorbitol (90 g/L), 2,4-D (0.5 mg/L), Naphthalene acetic acid (NAA) (1 mg/L) and 6-Benzylaminopurine (0.5 mg/L) and incubated for 10 days under dark conditions. After stress treatment, the calli were transferred to the plant regeneration induction medium, which consisted of N6 agar medium supplemented with hygromycin B (50 mg/L), glucose (5 g/L), NAA (0.5 mg/L) and kinetin (5 mg/L), and then incubated for 30 days to induce the formation of shoots and roots from the callus. The regenerated rice seedlings were then transferred to the 1/2 Murashige and Skoog (MS) medium for further growth. The transgenic rice seedlings (T0 plants) were finally transferred and grown in the soil pot for further studies and the transgenic seeds (T1 seeds) were harvested.

## Data Availability

The raw data supporting the conclusions of this article will be made available by the authors, without undue reservation.
