# Supplementary material for: Host-induced gene silencing targeting the calcineurin of Fusarium fujikuroi to enhance resistance against rice bakanae disease
Source: Front Plant Sci. 2025 Apr 14;16:1366158. doi: 10.3389/fpls.2025.1366158 (PMC12035443; doi:10.3389/fpls.2025.1366158)
Supplement: Supplementary file 1 [file DataSheet1.pdf]

**Supplementary information**

Hou and Chang *et al*., 2025. Host-induced gene silencing targeting the calcineurin of *Fusarium fujikuroi* to enhance resistance against rice bakanae disease.

**A** **B** **C**

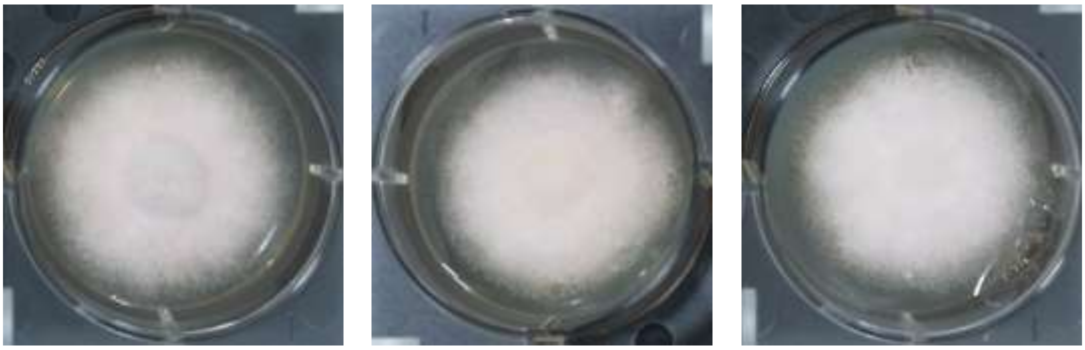

**D**

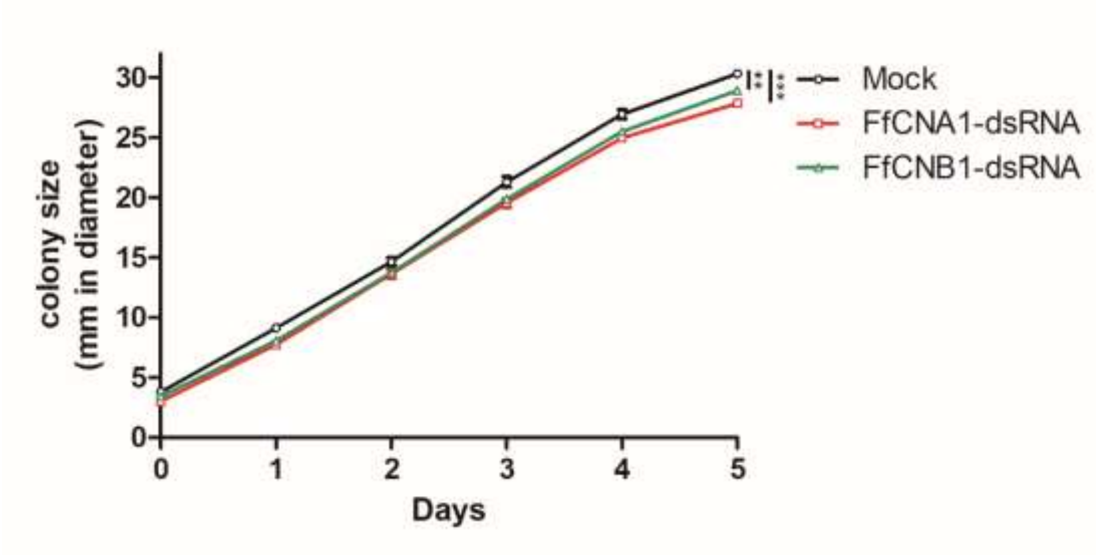


**Figure S1. Growth inhibition of *F. fujikuroi* by *in vitro* dsRNA application.** A single spore of *F. fujikuroi* IL01 was treated with **(A)** DEPC water (Mock), **(B)** *FfCNA1*-dsRNA, and **(C)** *FfCNB1*-dsRNA for 5 days to evaluate the efficacy of exogenous dsRNA. Droplets containing 1,500 ng of dsRNA were applied every 12 hours. **(D)** Growth kinetics analysis of *F. fujikuroi* IL01 following the treatments above. Error bars represent standard deviation. These results are obtained from six biological replicates. Asterisks indicated significant differences compared to wild-type according to *t*-test, **,*** indicates *P* value < 0.01, 0.001, respectively.

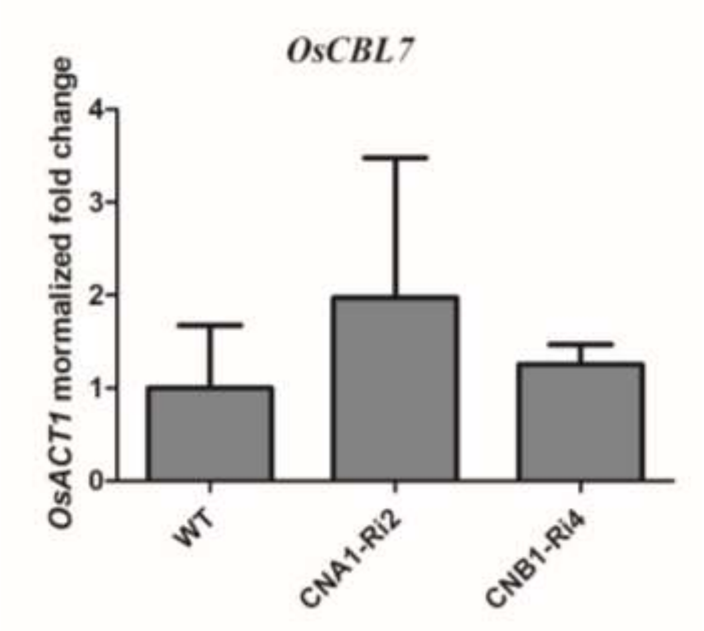


**Figure S2. The relative expression level of *OsCBL7* did not differ significantly among the rice lines.** Two-week-old rice seedlings infected with *F. fujikuroi* IL01 were used for RNA extraction and RT-qPCR. Total RNAs were extracted from rice seedlings with TRIzol reagent (Invitrogen, Carlsbad, CA, USA) according to the protocol. The extracted RNAs were treated with TURBO DNA-free kit (Invitrogen, USA), then perform cDNA synthesis with a high-capacity cDNA reverse transcription kit (Applied Biosystems, CA, USA). To analyze the effects caused by the presence of dsRNAs, quantitative-PCR was applied with SensiFAST$^{TM}$ SYBR Hi-ROX Mix (2x) (Biolin, USA) to evaluate the expression of *OsCBL7* in wild-type and transgenic rice lines. The expression level was normalized to the *O. sativa ACT1* gene using $2^{-\Delta\Delta Ct}$ method. No significant difference was found between wild-type and transgenic rice lines according to one-way ANOVA statistically analysis ($P > 0.05$).

**A**

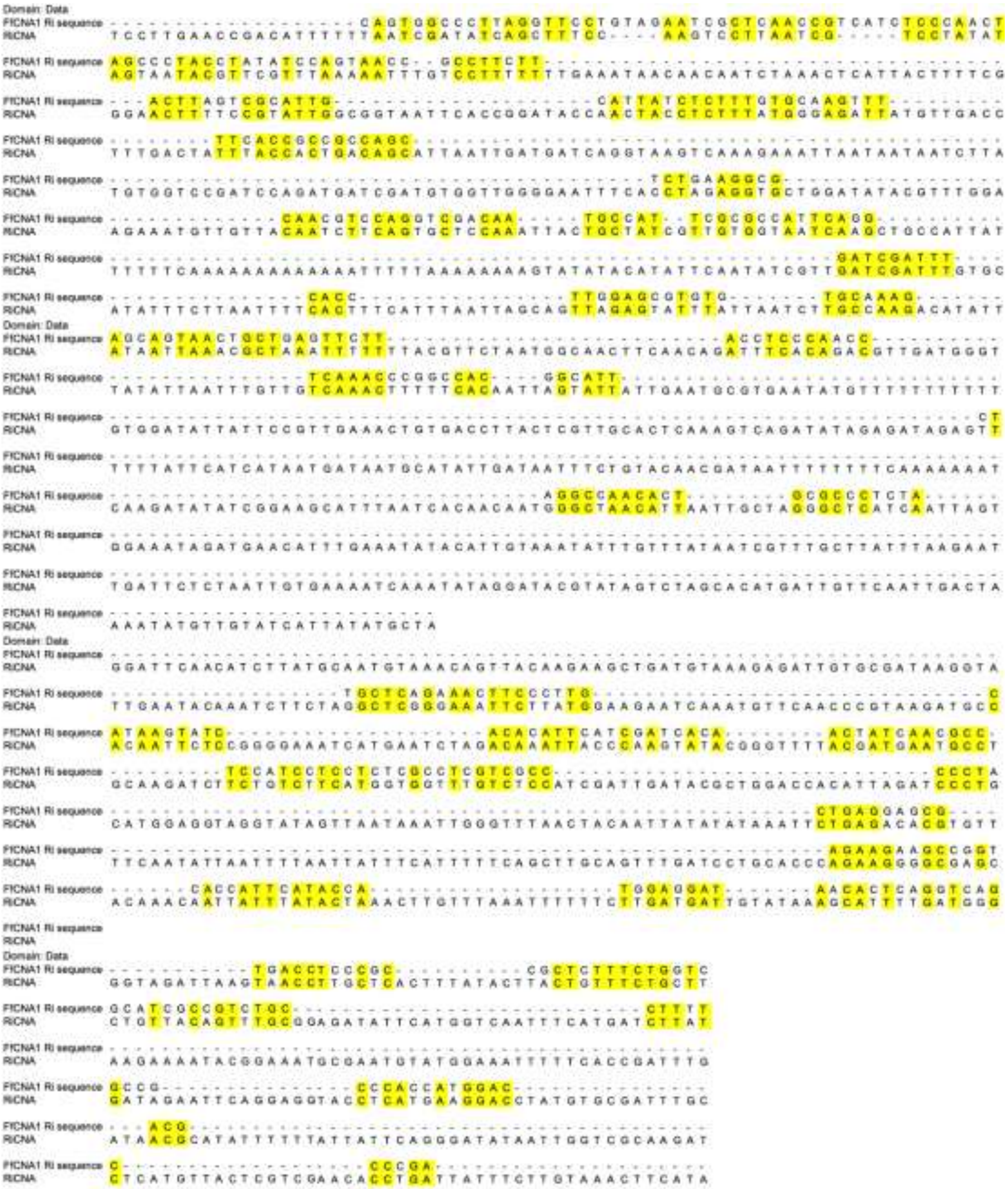

**B**

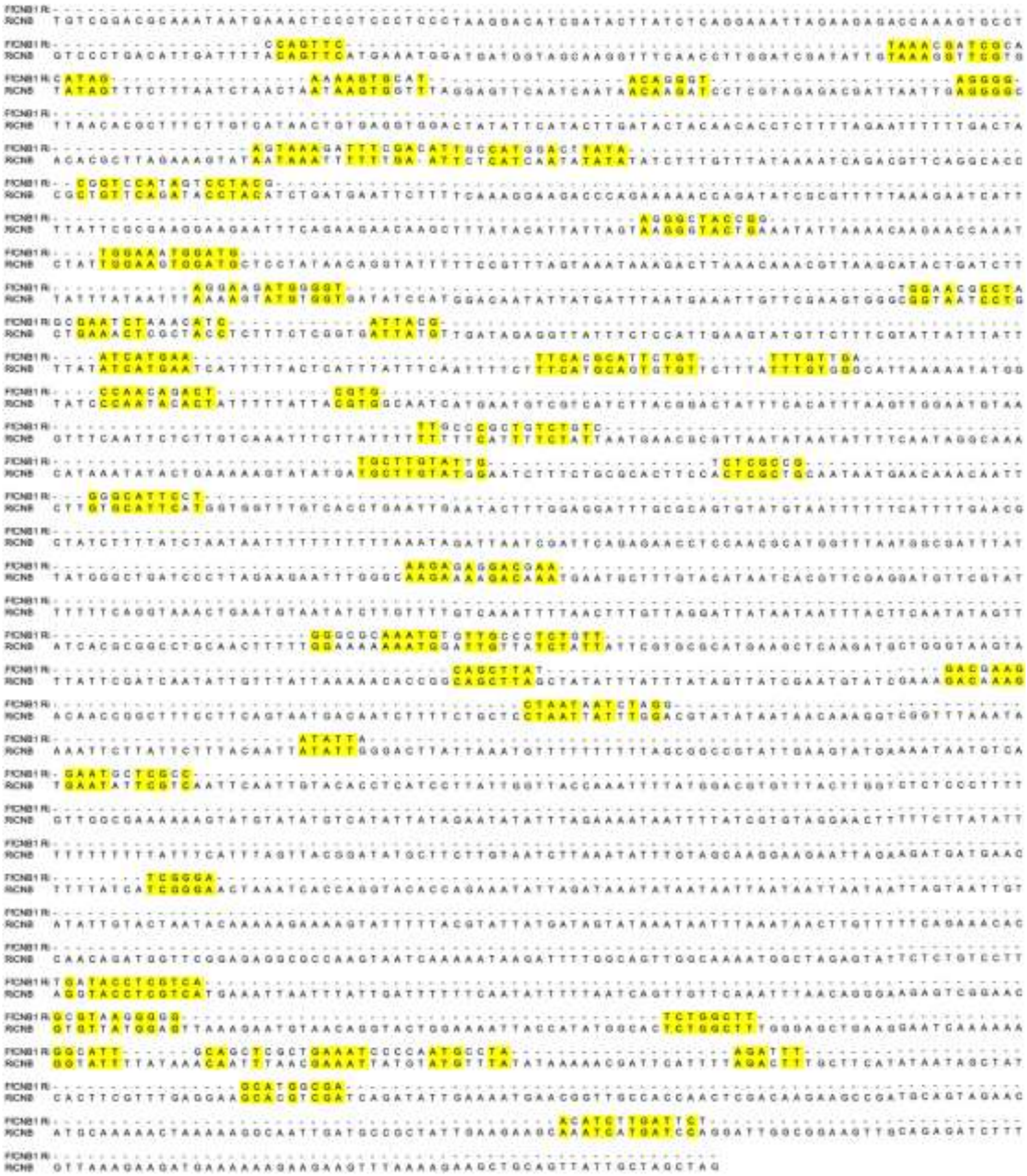

**Figure S3. Off-target prediction of *FfCNA1*-RNAi and *FfCNB1*-RNAi construct towards *Glomeraeae* sp.** Comparison of the nucleotide sequence used in *FfCNA1*-RNAi with the nucleotide sequence of calcineurin subunit A (**A**) and *FfCNB1*-RNAi with the nucleotide sequence of calcineurin subunit B (**B**) in *Rhizophagus irregularis* (NW_020269546.1). The shaded region indicated conserved residues. The sequence alignment was analyzed using MEGA software.

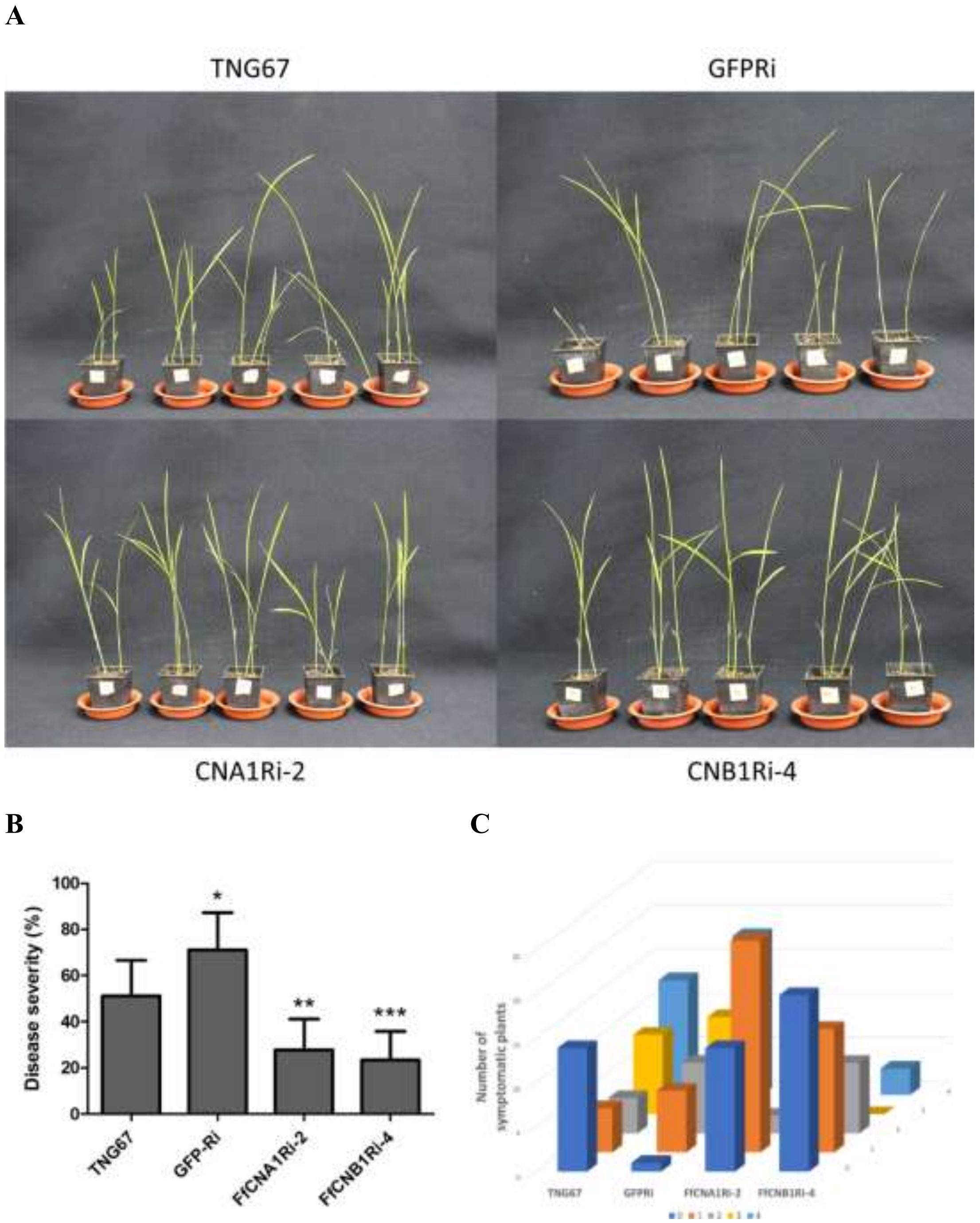


**Figure S4. Ectopic expression of GFP-dsRNA in transgenic rice has modestly impact on the virulence of *F. fujikuroi*.** Wild-type and transgenic rice lines are inoculated with *F. fujikuroi* IL01 by the method described in the section of Materials and Methods. **(A)** The phenotypes, **(B)** disease severity, and **(C)** disease grades were recorded at 21 dpi. Error bars represent standard deviation. These results were obtained from three biological replicates. Asterisks indicated significant differences compared to wild-type according to one-way ANOVA. *,**,*** indicates *P* values < 0.05, 0.01, and 0.001, respectively.

**A**

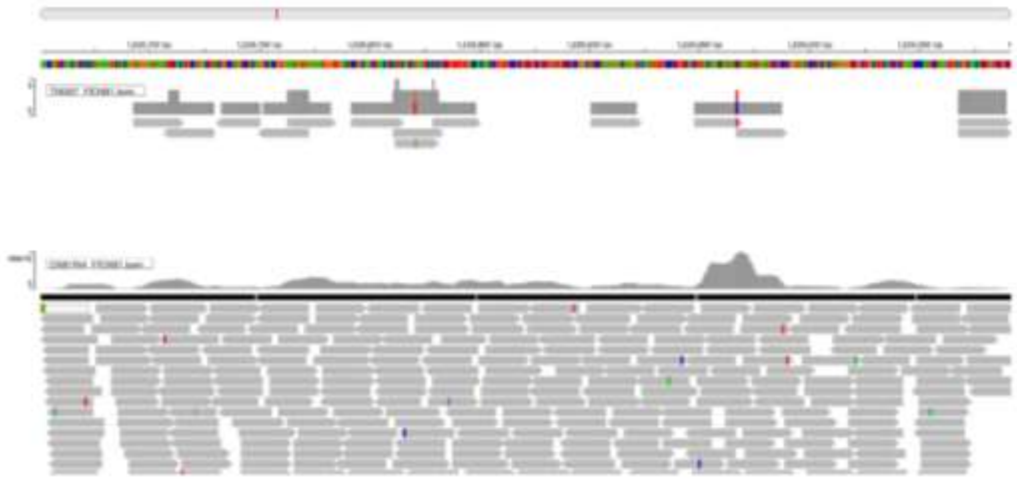

**B**

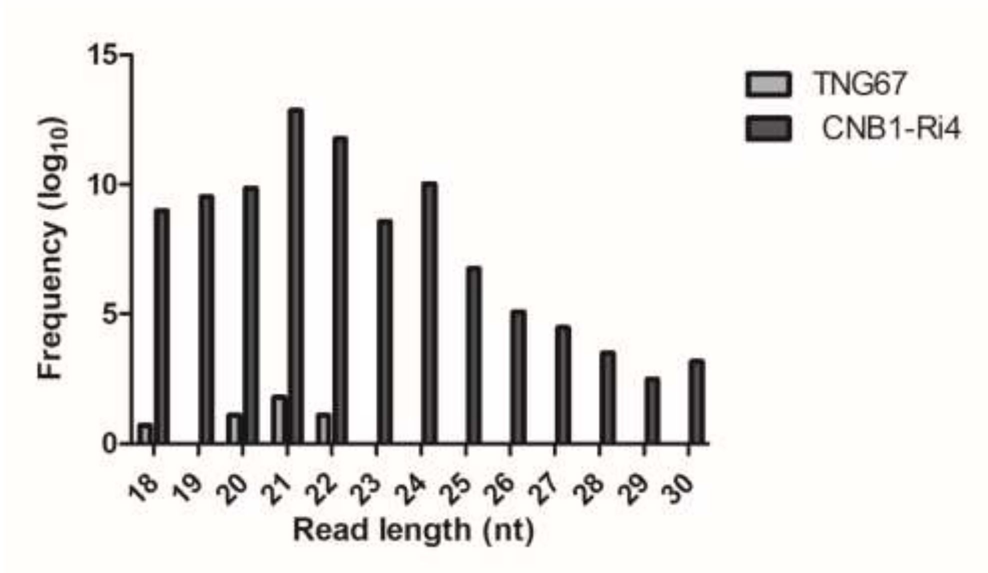


**Figure S5. The miRNAseq analysis of FfCNB1-derived RNAs in TNG67 and CNB1-Ri4.** Total RNAs from TNG67 and CNB1-Ri4 were extracted using miRNeasy mini kit (Qiagen, Valencia, CA, USA) according to the manufacturer's protocol. The siRNAs ranging from 18 to 30 nucleotides in length were selected and mapped to the genome of *F. fujikuroi* (GCF_900079805.1) using Bowtie 2 to analyze their distribution and sequence similarity to *FfCNB*1-dsRNA. **(A)** The distribution of mapped siRNAs was further verified using the IGV tool, and **(B)** the frequency was calculated.
